# *In vitro* anti-herpes simplex virus-2 activity of *Salvia desoleana* Atzei & V. Picci essential oil

**DOI:** 10.1371/journal.pone.0172322

**Published:** 2017-02-16

**Authors:** Valeria Cagno, Barbara Sgorbini, Cinzia Sanna, Cecilia Cagliero, Mauro Ballero, Andrea Civra, Manuela Donalisio, Carlo Bicchi, David Lembo, Patrizia Rubiolo

**Affiliations:** 1 Laboratory of Molecular Virology, Department of Clinical and Biological Sciences, Università degli Studi di Torino, Orbassano, Turin, Italy; 2 Dipartimento di Scienza e Tecnologia del Farmaco, Università degli Studi di Torino, Torino, Italy; 3 Dipartimento di Scienze della Vita e dell’Ambiente, Università degli Studi di Cagliari, Cagliari, Italy; Cornell University, UNITED STATES

## Abstract

*Salvia desoleana* Atzei & V. Picci is an indigenous species in Sardinia island used in folk medicine to treat menstrual, digestive and central nervous system diseases. Nowadays, it is widely cultivated for the pleasant smell of its essential oil (EO), whose antimicrobial and antifungal activities have already been screened. This study evaluated the *in vitro* anti-Herpes Simplex Virus-2 (HSV-2) activity of *S*. *desoleana* EO, fractions and main components: linalyl acetate, alpha terpinyl acetate, and germacrene D. Phytochemical composition of *S*. *desoleana* EO was studied by GC-FID/MS analysis and the active fraction(s) and/or compounds in *S*. *desoleana* EO were identified with a bioassay-guided fractionation procedure through *in vitro* assays on cell viability and HSV-2 and RSV inhibition. *S*. *desoleana* EO inhibits both acyclovir sensitive and acyclovir resistant HSV-2 strains with EC_50_ values of 23.72 μg/ml for the former and 28.57 μg/ml for the latter. Moreover, a significant suppression of HSV-2 replication was observed with an EC_50_ value of 33.01 μg/ml (95% CI: 26.26 to 41.49) when the EO was added post-infection. Among the fractions resulting from flash column chromatography on silica gel, the one containing 54% of germacrene D showed a similar spectrum of activity of *S*. *desoleana* EO with a stronger suppression in post-infection stage. These results indicated that *S*. *desoleana* EO can be of interest to develop new and alternative anti-HSV-2 products active also against acyclovir-resistant HSV-2 strains.

## Introduction

Genital herpes is a sexually transmitted disease most commonly caused by herpes simplex virus type 2 (HSV-2), a lipid-enveloped DNA virus belonging to the *Herpesviridae* family. After the primary infection, HSV-2 establishes a lifelong latent infection in the lumbosacral sensory ganglia. The reactivation can be triggered by several stimuli including immunosuppression, stress, and hormonal changes. As a result, asymptomatic episodes in which the virus can be spread or recurrent painful ulcerations to the genital mucosa may occur, which, in turn, increase the risk of human immunodeficiency virus acquisition [1–3]. Besides the sexual transmission, HSV-2 is correlated with a serious risk of mother to child infection during birth [4].

Currently, no vaccine is available but the antiviral drugs acyclovir, penciclovir and their derivates, valacyclovir, and famciclovir are effective treatments for herpes simplex infections [5,6]. These drugs are nucleoside analogues that interfere with viral DNA polymerization shortening the course of the illness and decreasing the severity of the symptoms but are not active in suppressing the latent infection. Moreover, due to their wide use, drug-resistant HSV strains are nowadays common, particularly in immunocompromised patients [7].

For these reasons, it is still important to identify new antiviral substances (against HSV-2 acting with a different mechanism than nucleoside analogues to override the drug-resistance. In this context, natural products could be rich sources of antiviral molecules due to their large structural diversity and complexity [8]. Indeed, both plant extracts and essential oils have been recently reported to inhibit HSV-2 infection [9–13].

*Salvia desoleana* Atzei & V. Picci [14,15] is an herbaceous perennial shrub belonging to Lamiaceae family. It is endemic and exclusive of the island of Sardinia (Italy), but it is widely cultivated because of its EO. The plant is 80–180 cm tall, with ovate leaves covered in both surface by hairs and glands, releasing a strong fragrance when crushed or brushed. The inflorescences (30–50 cm) have hairy stems, with evenly spaced whorls of six flowers. The flowers have a pale lavender upper lip, and an off-white trough-shaped lower lip. The plant blooms in May-July and the fruits develop in September-November.

In Sardinian traditional medicine, the leaves of *S*. *desoleana* were used in decoction as antipyretic, and in poultice and macerated as anti-inflammatory and to heal wounds [16].

A previous study showed a weak microbiostatic action of *S*. *desoleana* EO against *S*. *aureus*, *E*. *coli*, *S*. *epidermidis* and *C*. *albicans* [17], however, no antiviral activity has been reported yet. The present study was undertaken to test *S*. *desoleana* EO for its antiviral activity against HSV-2, to investigate its probable mode of action and to identify its active ingredient(s). Interestingly, among the pure compounds identified in the active fraction, several have been previously identified as inhibitors of HSV-1 infection [18–20].

Both the EO as such and an EO fraction enriched in its constituent germacrene D, proved to be active against acyclovir-sensitive and acyclovir-resistant viral strains suppressing HSV-2 replication after virus attachment/entry.

## Materials and methods

### Materials

1,8 cineole, linalool and linalyl acetate with a purity > 97%, terpinyl acetate (mixture of two isomers) with a purity > 95%; β-caryophyllene (mixture of two isomers) with a purity > 98.5%,decane (purity >99%) high purity silica gel 70–230 mesh and silver nitrate on silica gel (about 10% wt, % loading + 230 mesh) were from Sigma-Aldrich (Milan, Italy). HPLC and analytical grade solvents were from Carlo Erba Reagenti (Rodano, Italy). Commercially available standards were chosen to confirm component identification in the essential oil and to quantify the EO components on the basis of their response factor towards an internal standard (decane) considering the potential shift of FID towards the different classes of compounds [21]. Acyclovir, used as a positive control for HSV-2 antiviral activity was purchased from Sigma-Aldrich (Milan, Italy).

### Essential oil (EO)

The aerial parts of *S*. *desoleana* (20 Kg) were harvested during the flowering period from Sadali (CA), Sardinia (Italy) and derived from plants cultivated by the Consortium Fragus e saporis de Sardinia. The Consortium Fragus e saporis de Sardinia gave permission to conduct the study on this site. The taxonomical identification was performed by Dr. Cinzia Sanna and a voucher specimen (Herbarium CAG 1086) has been retained in the General Herbarium of the botanical garden of the University of Cagliari. EO from fresh plant materials was obtained in agreement with the European Pharmacopoeia VIII ed.: fresh crushed aerial parts were submitted to steam distillation for five hours (h). The resulting EO were left to stabilize for one hour. The yield in essential oil was 0.12% (v/w on fresh material).

### Essential oil fractionation

*S*. *desoleana* EO (1.2 g) was fractionated on silica gel column chromatography using petroleum ether with increasing amounts of ethyl acetate. Two main fractions (SD1 and SD2) were obtained with 100% petroleum ether and 80/20 petroleum ether/ethyl acetate respectively. Fraction SD1 represents the 25% of the total EO and mainly containing mono and sesquiterpene hydrocarbons. Fraction SD2, representing 59% of the total essential oil, mainly contained oxygenated mono and sesquiterpenoids.

### GC-FID-MS and enantioselective (ES)-GC-FID-MS analysis

GC analyses were carried out on a Shimadzu 2010 GC-FID system and a Shimadzu QP2010 plus GC-MS system, provided with an AOC-20i automatic injector. Data were processed with Shimadzu GC Solution 2.53SU1 software and Shimadzu1224 PGCMS Solution 2.51 software, respectively (Shimadzu, Milan, Italy).

GC-FID-MS analyses were carried out on a Mega 5 column (95% polydimethyl-siloxane, 5% phenyl) 25 m×0.25 mm dc ×0.25 μm df, from MEGA (Milan, Italy).

Analysis conditions were as follows: injection mode: split; split ratio: 1:20; injection volume: 1 μl. Temperatures: injector:250°C, FID: detector: 280°C, MS: transfer line:280°C; ion source: 200°C; carrier gas: He, initial flow rate 1.0 ml/min in constant linear velocity mode. The temperature programme was from 50°C (1 min) to 250°C (10 min) at 3°C/min (5°C/min for Mega-Dex DMT-Beta column). The MS was operated in electron impact ionization mode at70 eV, with a mass range of 35–350 m/z in full scan mode.

EO components were identified by comparison of both their linear retention indices (*I*^T^s), calculated versus a C9-C25 hydrocarbon mixture, and their mass spectra to those of authentic samples, or to those reported in commercially available mass spectral libraries [22].

### Cells

African green monkey fibroblastoid kidney cells (Vero, ATCC CCL-81), the epithelial cell lines Hep-2 (*ATCC* CCL-23) and A549 (*ATCC* CCL-185) were grown as monolayers in Eagle’s minimal essential medium (MEM) (Gibco/BRL, Gaithersburg, MD) supplemented with 10% heat inactivated fetal calf serum (FCS) and 1% antibiotic-antimycotic solution (Zell Shield, Minerva Biolabs GmbH, Berlin, Germany).

### Viruses

A clinical isolate of HSV-1 and HSV-2 were kindly provided by Prof. M. Pistello, University of Pisa, Italy. HSV strains was propagated and titrated by plaque assay on Vero cells. A HSV-2 strain with phenotypic resistance to acyclovir was generated by serial passage in the presence of increasing concentrations of acyclovir, as previously described [23]. Virus stocks were maintained at -80°C. Respiratory syncytial virus (RSV) strain A2 (ATCC VR-1540) was propagated in Hep-2 cells by infecting a freshly prepared confluent monolayer grown in MEM supplemented with 2% of FCS. When the cytopathic effect involved the whole monolayer, the infected cell suspension was collected and the viral supernatant was clarified. The virus stocks were aliquoted and stored at -80°C. The infectivity of virus stocks was determined on Hep-2 cell monolayers by standard plaque assay.

### Cell viability

Cell viability was measured using the MTS [3-(4,5-dimethylthiazol-2-yl)-5-(3-carboxymethoxy- phenyl)-2-(4-sulfophenyl)-2H-tetrazolium] assay, as described by Civra et al. [24]. Confluent cell cultures seeded in 96-well plates were incubated with serially diluted EO, fractions, constituents or acyclovir in triplicate under the same experimental conditions described for the antiviral assays. The effect on cell viability at different concentrations was expressed as a percentage, by comparing absorbances of treated cells with those of cells incubated with culture medium supplemented of equal volume of DMSO. The 50% cytotoxic concentrations (CC_50_) and 95% confidence intervals (CIs) were determined using Prism software (Graph-Pad Software, San Diego, CA).

### HSV inhibition assays

The effect of *S*. *desoleana* EO, fractions, constituents or acyclovir on HSV infection was evaluated by plaque reduction assay. Vero cells were pre-plated 24 h in advance in 24-well plates at a density of 10 x 10^4^ cells. Increasing concentrations of EO, fractions, constituents or acyclovir were added to cells for two hours; a mixture of the different tested substances plus HSV-2 or HSV-1 or acyclovir resistant HSV-2 (MOI 0.0005 pfu/cell) was subsequently added to the cells, which were then incubated at 37°C for two hours. The virus inoculum was then removed and the cells washed and overlaid with a medium containing 1.2% methylcellulose (Sigma) and serial dilutions of EO, fractions, constituents or acyclovir. For acyclovir antiviral assay the compound was added only after infection, diluted in the medium containing methylcellulose as described before. After further incubation at 37°C for 24 hours, cells were fixed and stained with 0.1% crystal violet in 20% ethanol and viral plaques counted. The concentration producing 50% reduction in plaque formation (EC_50_) was determined using Prism software by comparing drug-treated with untreated wells. Three independent experiments were performed in duplicate.

Moreover, the plaques were visualized using a Leica inverted microscope equipped with a Bresser MikroCam microscope camera and MikroCamLab software (Rhede, Germany). ImageJ software was used to quantify plaque sizes. Untreated HSV2-infected monolayers were used as the control.

### RSV inhibition assay

To evaluate the ability to inhibit RSV infection, EO was serially diluted to generate dose-response curves and added to cells. After 2 h, EO was removed and a mixture of EO dilutions and RSV (MOI 0.01 PFU/cell) was added to A549 cells grown as monolayers in a 96-well plate at a density of 7 x 10^4^/well. After 3 hours of incubation at 37°C, monolayers were washed and overlaid with 1.2% of methylcellulose medium containing serial dilutions of EO. Three days post-infection, cells were fixed with cold methanol and acetone for 1 min and subjected to RSV-specific immunostaining using an RSV monoclonal antibody (Ab35958, Abcam, Cambridge, UK) and the UltraTech HRP streptavidin-biotin detection system (Beckman Coulter, Marseille, France). Immunostained syncytia were counted and the percent inhibition of virus infectivity determined by comparing the number of plaques in treated wells with the number in untreated control wells. 50% effective concentration (EC50) values and 95% CIs were determined using Prism software. All data were generated from duplicate wells in at least three independent experiments.

### Time-of-addition assay

Serial dilutions of EO or fraction SD1 were added to cells: two hours before infection (pre-treatment assay), during infection, or post infection (post-treatment assay). After the incubation time, viral plaques were counted.

### Virus inactivation assay

Approximately 10^5^ PFU of HSV-2 plus EC90 of *S*. *desoleana* EO was added to MEM and mixed in a total volume of 100 μl. The virus-EO mixtures were incubated for zero hours or two hours at 37°C then diluted serially to the non-inhibitory concentration of *S*. *desoleana* EO; the residual viral infectivity was determined by viral plaque assay.

### Data analysis

All results are presented as the mean values from three independent experiments performed in duplicate. The EC_50_ values for inhibition curves were calculated by regression analysis using the software GraphPad Prism version 4.0 (GraphPad Software, San Diego, California, U.S.A.) by fitting a variable slope-sigmoidal dose—response curve. The selectivity index (SI) was calculated by dividing the CC_50_ by the EC_50_ value.

## Results and discussion

Within a project aiming to investigate the potential antiviral activity of endemic plants from Sardinia, this study evaluated the anti-HSV-2 activity of *S*. *desoleana* EO, fractions and its main components. The EO was obtained by steam distillation of the fresh aerial part from cultivated plants in order to obtain a more standardized product. Table 1 reports its chemical composition. Thirty-four components were identified accounting for 95.8% of the EO and the main components were linalyl acetate (30.2%), germacrene D (18.7%) and alpha terpinyl acetate (16.8%). The qualitative EO composition here reported is coherent with those reported in the literature and affords to differentiate *S*. *desoleana* from *S*. *sclarea* [17,25,26]. *S*. *desoleana* EO composition was confirmed by the distillation of plants in different years from 2010 to 2013 (data not shown).

**Table 1:** Chemical composition (expressed as g/100g) of *S*. *desoleana* essential oil and its fractions.

#	Compounds	*I*^t^*s*_*exp*_^a^	*I*^t^*s*_*lit*_^b^	EO	SD1 fraction	SD2 fraction
1	α-thujene	929	931	0.2	0.3	
2	α-pinene	936	939	0.5	0.5	
3	sabinene	975	976	1.0	1.3	
4	β-pinene	977	980	1.2	2.7	
5	myrcene	992	991	1.3	2.3	
6	limonene	1028	1031	1.0	1.8	
7	1,8-cineole	1033	1033	10.2		12.6
*8*	*cis*-β-ocimene	1042	1040	1.4	3.3	
*9*	*trans*-β-ocimene	1052	1050	0.5	1.1	
10	γ-terpinene	1061	1062	tr	0.2	
11	α-terpinolene	1088	1088	0.2	0.5	
12	linalool	1101	1098	5.1		8.1
13	terpinen-4-ol	1177	1177	0.2		0.4
14	α-terpineol	1190	1189	1.8		0.2
15	linalyl acetate	1260	1257	27.3		39.7
16	α-terpinyl acetate	1352	1350	17.5		30.1
*17*	neryl acetate	1367	1365	tr		0.1
18	α-copaene	1377	1376	0.7	1.6	
19	β-bourbonene	1383	1384	0.3	0.9	
20	geranyl acetate	1385	1383	tr		1.0
21	β-elemene	1392	1391	1.0	2.7	
22	β-caryophyllene	1415	1418	1.3	4.8	
23	aromadendrene	1437	1439	0.4	1.3	
24	germacrene D	1479	1480	17.9	55.0	
25	bicyclogermacrene	1492	1494	0.2	3.1	
26	γ-cadinene	1512	1513	0.4	1.3	
27	δ-cadinene	1522	1524	0.3	1.3	
28	MW = 222	1613	/	0.2		0.5
29	MW = 222	1638	/	0.6		1.3
30	β-eudesmol	1648	1649	0.6		0.6
31	MW = 272	1928	/	tr	0.7	
32	MW = 272	1953	/	1.6	6.7	
33	MW = 272	1966	/	0.4	2.1	
34	MW = 272	1980	/	0.3	0.7	
	**Total**			95.8	96.2	96.5

^**a**^ ***I***^**T**^***s***_***exp***_: experimental linear retention index

^**b**^***I***^**T**^**s**_***lit***_: linear retention index from literature

tr: amount below 0.1g/100g

The EO anti-HSV-2 activity, was initially investigated by a complete protection assay in which increasing EO concentrations were added to the cell culture before, during, and after the infection to generate dose-response curves. Acyclovir was tested in parallel with EO as a reference drug. As shown in Table 2, the EO EC_50_ value was equal to 23.72 μg/ml whereas the CC_50_ was 1577 μg/ml with a SI of 66.48, indicating that the antiviral effect was not a consequence of cytotoxicity. Of note, the EO antiviral activity was found to be independent on the virus sensitivity to acyclovir as demonstrated by the EC_50_ value of 28.57 μg/ml against an acyclovir-resistant HSV-2 (Table 3) which is similar to that calculated for the acyclovir-sensitive strain (Table 2).

**Table 2:** Antiviral activity against HSV-2.

	EC_50_^a^ 95% CI^b^ (μg/ml)	CC_50_^c^ (μg/ml)	SI^d^
Total Essential oil	23.72 (16.09–34.97)	1577	66.48
SD1	10.69 (8.36–13.68)	58.11	5.44
SD2	n.a.^e^	358.2	n.a.
β-Caryophyllene	5.38 (3.42–8.47)	49.00	9.10
Terpinyl acetate	n.a.	507.5	n.a.
Linalyl acetate	n.a.	1014	n.a.
Linalool	n.a.	989.6	n.a.
1,8-Cineole	n.a.	>1710	n.a.
Acyclovir	0.14 (0.098–0.21)	165	1178

^a^EC50: effective concentration 50%

^b^CI: confidence interval

^c^CC50: cytotoxic concentration 50%

^d^SI: selectivity index

^e^n.a.: not assessable

**Table 3:** Antiviral activity against HSV-2 acyclovir resistant.

	EC_50_^a^ 95% CI^b^ (μg/ml)	CC_50_^c^ (μg/ml)	SI^d^
Essential oil	28.57 (18.90–29.76)	1577	55.20
SD1	6.58 (5.79–7.48)	58.11	8.83
β-Caryophyllene	5.02 (3.55–7.10)	49.00	9.76
Acyclovir	71.84 (41.3–103.4)	165	2.29

^a^EC50: effective concentration 50%

^b^CI: confidence interval

^c^CC50: cytotoxic concentration 50%

^d^SI: selectivity index

^e^n.a.: not assessable

The activity against an acyclovir-resistant HSV-2 strain suggests that the EO mode of action differs from that of acyclovir, an interesting feature in light of a possible development of the EO as an anti-HSV-2 treatment. Despite of antiviral activity of EO against HSV-2 infection, no inhibitory activity was observed against the other member of *Herpesviridae* family, HSV-1 (data not shown).

Next, the EO was tested against RSV, another lipid-enveloped virus belonging to a different virus family (*Paramyxoviridae*) as HSV-2, to assess the specificity of EO antiviral action. Interestingly, we could not observe any inhibition demonstrating that the EO antiviral activity is virus-specific (data not shown).

**Fig 1:**
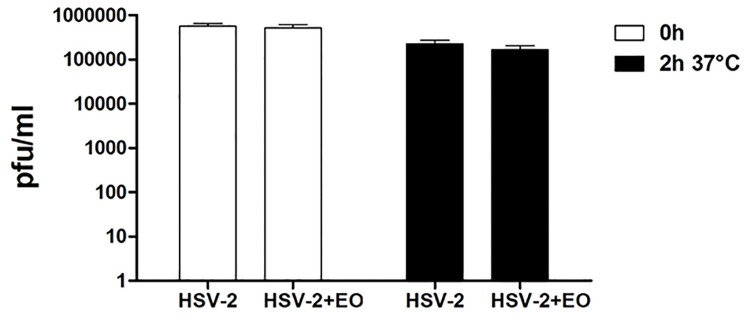
Virus inactivation assay 10^5^ pfu of HSV-2 were incubated with 190 μg/ml of *S*. *desoleana* EO for 0 or 2 h at 37°C. The mixtures were then titrated on Vero cells at high dilutions at which the concentration of EO was not active. The titers, expressed as pfu/ml, are means and SEM for 3 independent experiments performed in duplicate.

To explore whether the EO directly inactivates HSV-2 particles, a virus inactivation assay was performed with a not cytotoxic concentration of EO that reduces almost completely virus infection (EC_90_). HSV-2 aliquots were therefore incubated with 190 μg/ml of EO for 0 hours or 2 hours at 37°C. After incubation, the samples were titrated on Vero cells at high dilutions, at which residual EO was no longer active. As reported in Fig 1, this treatment did not produce a significant loss of HSV-2 infectivity demonstrating that EO does not exert any direct effect on viral particles.

**Fig 2:**
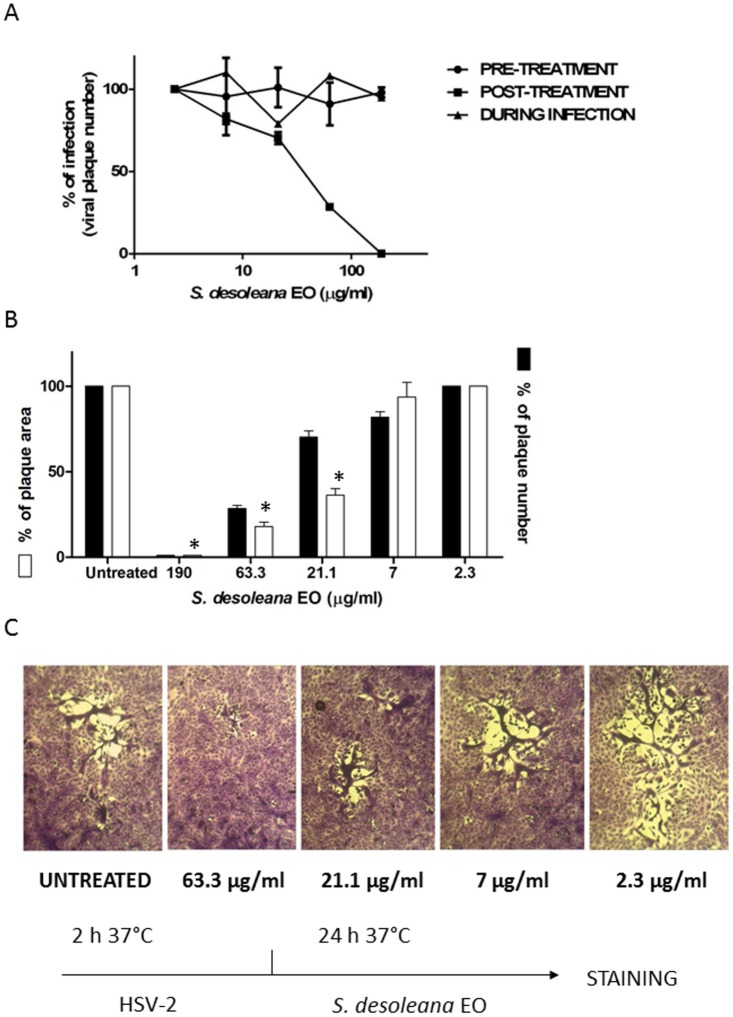
Time-of-addition assays with *S*. *desoleana* EO. A) Vero cells were treated with EO prior to virus infection (pre-treatment), during the infection period (during infection), or after infection (post-treatment). Data are presented as % of control. Values are means ± SEM of three independent experiments performed in duplicate. B) The histograms show the percentage of plaque area and plaque number of treated wells compared to that of untreated wells as a function of the concentration in the post-treatment assay. C) The images show representative plaques in Vero cells. The pictures and histograms shown are representative of many analyzed plaques, ranging from 15 to 25 per condition. * P< 0.0001.

This result indicates that the EO most probably targets cell-surface or intracellular events involved in essential steps of the HSV-2 replicative cycle. Therefore, time-of-addition assays was used to investigate which step(s) of the virus replicative cycle were affected by the EO (Fig 2). Since some antiviral compounds are known to lower cell susceptibility to viral infection by downregulating or directly masking virus receptors, the EO was tested by a pre-treatment assay. The results, deriving from three independent experiments, showed that different doses of EO added 2 hours prior to virus infection and then washed out before virus inoculum did not exert any inhibitory activity. Moreover, no inhibition was observed when the EO was added during infection ruling out an inhibitory effect on virus attachment and/or entry. In contrast, when it was added just after the virus inoculum and left throughout the assay, a significant suppression of HSV-2 replication was observed with an EC_50_ value of 33.01 μg/ml. Interestingly, the EO not only significantly reduced the plaque number but also decreased the area of the remaining plaques (Fig 2B and 2C).

Taken together, the results from the virus inactivation and time-of-addition assays indicate that EO exerts its antiviral activity by inhibiting later steps of the virus replicative cycle (e.g. uncoating, genome replication, virus assembly or exit, cell-to-cell spread). This effect can be ascribable to several EO components acting together or to a single chemical species. To assign the antiviral activity to specific fractions and/or to single components of *S*. *desoleana* EO a bioassay-guided fractionation procedure was adopted. Fraction SD1 was obtained with flash column chromatography on silica gel eluted with petroleum ether (100%). This fraction was characterized by mono and sesquiterpene hydrocarbons where germacrene D was the most abundant terpene hydrocarbon representing the 54% of the fraction; the second more abundant component was beta-caryophyllene (4.8% of SD1 fraction). Fraction SD2 (80/20 petroleum ether/ethyl acetate) was characterized by the presence of a mixture of oxygenated monoterpenes the most abundant are linalyl acetate, α-terpinyl acetate, 1,8 cineole and linalool (Table 1). The two fractions SD1 and SD2 were tested along with the pure commercially available standards of 1,8 cineole, linalool, linalyl acetate and α-terpinyl acetate and β-caryophyllene.

As shown in Table 2, SD1 was found to be the only active fraction with an EC_50_ of 10.69 μg/ml; Neither SD2 nor the pure oxygenated monoterpenes tested exerted any antiviral activity.

**Fig 3:**
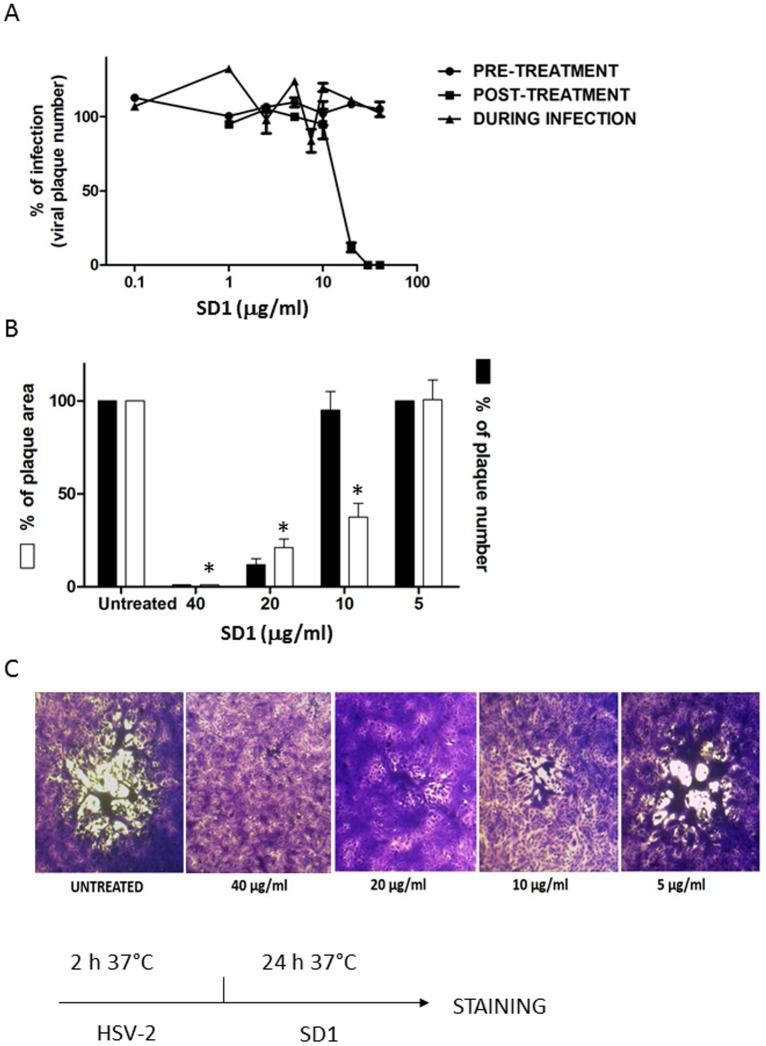
Time-of-addition assays with SD1 fraction. A) Vero cells were treated with SD1 fraction prior to virus infection (pre-treatment), during the infection period (during infection), or after infection (post-treatment). Data are presented as % of control. Values are means ± SEM of three independent experiments performed in duplicate. B) The histograms show the percentage of plaque area and plaque number of treated wells compared to that of untreated wells as a function of the concentration in the post-treatment assay. C) The images show representative plaques in Vero cells. The pictures and histograms shown are representative of many analyzed plaques, ranging from 15 to 25 per condition. * P< 0.0001.

SD1 fraction showed the same spectrum of activity of the EO not only against HSV-2, but also against the acyclovir-resistant strain with an EC_50_ of 6.58 μg/ml (Table 3) and it was no active against HSV-1 infection (data not shown). Furthermore, SD1 was active only when added to the cells after virus inoculum in time-of-addition assays (Fig 3A) and, performing post-treatment assay, it reduced the plaque number as well as the area of the remaining plaques (Fig 3B and 3C) as previously observed with the EO (Fig 2B and 2C). These data suggested that the antiviral effect of EO is totally due to SD1. Furthermore, SD1 inhibited viral infection in post-treatment assay with lower value of EC_50_ (16 μg/ml) rather than the value of EC_50_ of EO (33.01 μg/ml).

Interestingly, the pure commercially available standard of β-caryophyllene, a component of SD1 fraction, inhibits both HSV-2 and acyclovir-resistant strain infections with similar lower SI (9.10 and 9.76, respectively) in comparison with the EO (5.44).

Although the anti-HSV-2 activity of β-caryophyllene, we cannot exclude an antiviral role of other components of SD1, present in higher amount as germacrene D or present in lower quantities. Among the pure compounds identified in SD1 fractions, α-pinene, β-pinene, γ-terpinene, limonene, and β-caryophyllene have been previously identified as inhibitors of HSV-1 infection by direct interaction with free virus particles [18–20]. However, other papers did not evidence antiviral activity for α-pinene and β-caryophyllene against HSV-1 [27,28]. In accordance with these papers, we also not evidenced antiviral activity for β-caryophyllene against HSV-1 infection (data not shown). Moreover, β-myrcene, another component of SD1 fraction, was already shown to be devoid of antiviral activity against HSV-1 infection [27,28]. Germacrene D is a common component of several essential oils but, to the best of our knowledge, only one study concerning an ethanol extract of *Cedrus libani* leaves, containing a high percentage of germacrene D, reports results on tests against HSV-1 [27]. In fraction SD1, Germacrene D was the most abundant terpene hydrocarbon, however it is actually not available as commercial standard therefore further studies will be conducted to try to isolate it to verify if it may be considered potentially active against HSV-2.

## Conclusions

In conclusion, *S*. *desoleana* EO and SD1 fraction were here shown to be active against HSV-2 inhibiting a step of its replicative cycle that occurs after virus attachment and entry. Further studies are required to elucidate the mechanism of antiviral action and to identify the cellular and/or viral targets.

Anyway, the antiviral activity of *S*. *desoleana* EO and of the SD1 fraction against an acyclovir-resistant HSV-2 strain is an additional interesting feature prompting further studies to assess their potential in *in vivo* examinations.
